# Supplementary material for: Type 2 diabetes remission and its predictors in an Indian cohort: A retrospective analysis of an intensive lifestyle intervention program
Source: PLoS One. 2025 Oct 22;20(10):e0333114. doi: 10.1371/journal.pone.0333114 (PMC12543109; doi:10.1371/journal.pone.0333114)
Supplement: S1 Fig — (PDF) [file pone.0333114.s001.pdf]

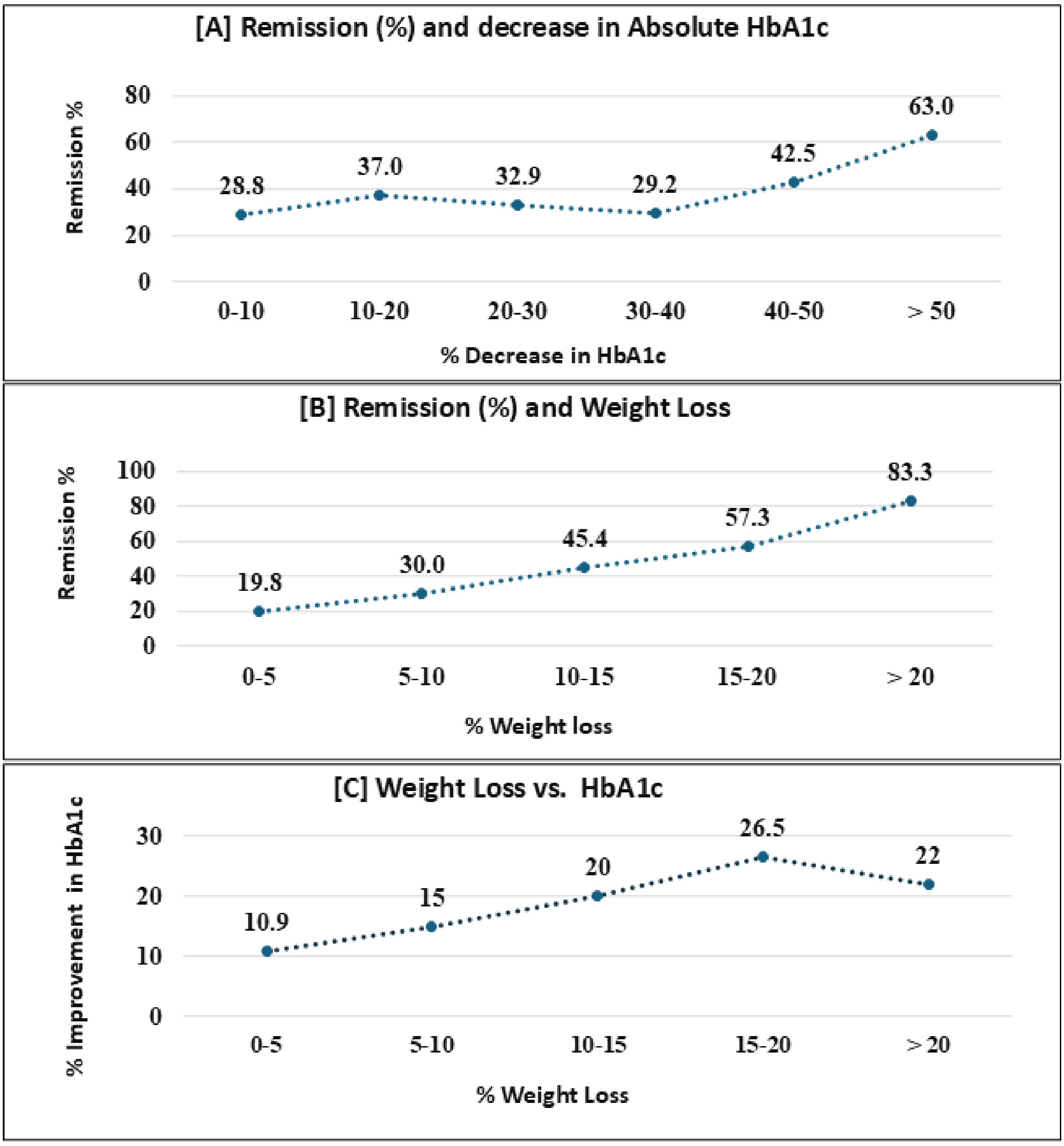

Supplemental Figure 1. Association of Remission with % reduction in HbA1c [A] and % weight loss [B] and Association between weight loss and % change in HbA1c [C]
